# Activation of Neuronal Nicotinic Receptors Inhibits Acetylcholine Release in the Neuromuscular Junction by Increasing Ca^2+^ Flux through Ca_v_1 Channels

**DOI:** 10.3390/ijms22169031

**Published:** 2021-08-21

**Authors:** Nikita Zhilyakov, Arsenii Arkhipov, Artem Malomouzh, Dmitry Samigullin

**Affiliations:** 1 Kazan Institute of Biochemistry and Biophysics, FRC Kazan Scientific Center, Russian Academy of Sciences, P.O. Box 261, 420111 Kazan, Russia; senjaarh@rambler.ru (A.A.); artur57@gmail.com (A.M.); 2 Department of Radiophotonics and Microwave Technologies, Federal State Budgetary Educational Institution of Higher Education “Kazan National Research Technical University Named after A.N. Tupolev–KAI”, 420111 Kazan, Russia

**Keywords:** neuromuscular junction, neurotransmitter release, acetylcholine, nicotinic receptor, calcium channel, calcium transient

## Abstract

Cholinergic neurotransmission is a key signal pathway in the peripheral nervous system and in several branches of the central nervous system. Despite the fact that it has been studied extensively for a long period of time, some aspects of its regulation still have not yet been established. One is the relationship between the nicotine-induced autoregulation of acetylcholine (ACh) release with changes in the concentration of presynaptic calcium levels. The mouse neuromuscular junction of m. Levator Auris Longus was chosen as the model of the cholinergic synapse. ACh release was assessed by electrophysiological methods. Changes in calcium transients were recorded using a calcium-sensitive dye. Nicotine hydrogen tartrate salt application (10 μM) decreased the amount of evoked ACh release, while the calcium transient increased in the motor nerve terminal. Both of these effects of nicotine were abolished by the neuronal ACh receptor antagonist dihydro-beta-erythroidine and Ca_v_1 blockers, verapamil, and nitrendipine. These data allow us to suggest that neuronal nicotinic ACh receptor activation decreases the number of ACh quanta released by boosting calcium influx through Ca_v_1 channels.

## 1. Introduction

Acetylcholine (ACh) is the main neurotransmitter in the peripheral nervous system of vertebrates and humans. In particular, it is responsible for the transmission of signals from the motor nerve to the skeletal muscles [1,2]. Since the neuromuscular junction is a key linker in the initiation of any motor act (from voluntary movement of the limbs, to breathing, to contraction of the vocal cords), an investigation of the regulation of neuromuscular transmission is of great importance for both fundamental neurobiology and applied medicine.

Since the midst of the 20th century, the data began to accumulate indicating that ACh, released in the synaptic cleft from the nerve endings, activates presynaptic cholinergic receptors, thus exerting a modulatory effect on the neurotransmission process by changing the amount and/or dynamics of subsequent portions of neurotransmitter release [2,3,4,5,6,7]. Initially pharmacologically, and later by other methods it has been shown that both ionotropic nicotinic and metabotropic muscarinic cholinergic receptors are present in the motor nerve terminal, and the activation of these receptors can lead to autoregulation of ACh release [4,5,8,9,10,11]. According to the data of morphological and functional analysis, presynaptic cholinergic receptors can be located both near the active zones and relatively far from the synaptic cleft [4,12,13].

When studying autoregulation mediated by muscarinic cholinergic receptors, it was found that the activation of the M1-subtype receptors led to facilitation of the release. In contrast, activation of the M2-subtype caused inhibition of the ACh quanta release [14,15]. Both the M1- and M2-mediated mechanisms depend on calcium influx [14,16,17,18].

Studies of the mechanisms of the autoregulation of ACh release mediated by nicotinic cholinergic receptors are complicated by the fact that the predominant population of these proteins is located in the postsynaptic membrane. Their activation is accompanied by the depolarization of sarcolemma and the subsequent generation of action potential, which ultimately leads to muscle contraction. The data indicate that the activation of presynaptic nicotinic cholinergic receptors leads to inhibition of the process of ACh release [19,20,21].

Also, experimental evidence was obtained indicating the possible involvement of voltage-gated calcium channels (VGCCs) of the L-type (Ca_v_1) in the modulation of neurotransmission [20,22]. Meanwhile, the results of a number of studies demonstrate that neither the Ca_v_1 type nor the N-type (Ca_v_2.2) VGCCs participate in the evoked release of ACh in mammalian neuromuscular contacts [23,24,25,26].

Thus, the question of the role of calcium channels in the mechanisms of the regulation of ACh release, as mediated by nicotinic cholinergic receptors, remains open as of now.

In the present study, using a pharmacological approach, electrophysiological techniques and the method of the optical registration of changes in calcium levels in the motor nerve endings, we made the following observations. An agonist of nicotinic receptors (at a concentration not significantly affecting the state of the postsynaptic membrane) leads to a decrease in the amount of released ACh quanta. This effect is accompanied not by a decrease, but by an increase of calcium ion entry into the motor nerve terminal. Our data suggest that the nicotinic cholinergic receptors responsible for the mechanism of ACh release autoregulation are the receptors of neuronal type. Activation of these receptors leads to upregulation of the Ca_v_1 type of VGCCs, resulting in the enhancement of Ca^2+^ entry into the nerve ending.

## 2. Results

### 2.1. Effects of Nicotine on the Electrophysiological Parameters of the Neuromuscular Junction

Using the intracellular microelectrode technique, we recorded the resting membrane potential (RMP) of the muscle fiber, amplitude of miniature endplate potentials (mEPPs), frequency of occurrence of mEPPs, and the amplitude of the evoked potentials of the end plate (EPPs).

Alterations in RMP and mEPP amplitude indicate the postsynaptic action of the pharmacological agent. Changes in the frequency of occurrence of mEPPs suggest the presynaptic action of the drug. The EPP amplitude, in turn, can vary due to changes at both the pre- and post-synaptic levels. Therefore, it was necessary to assess the effect of nicotine on every parameter mentioned above to determine the optimal effective concentration of nicotine to study the autoregulation.

**Figure 1:**
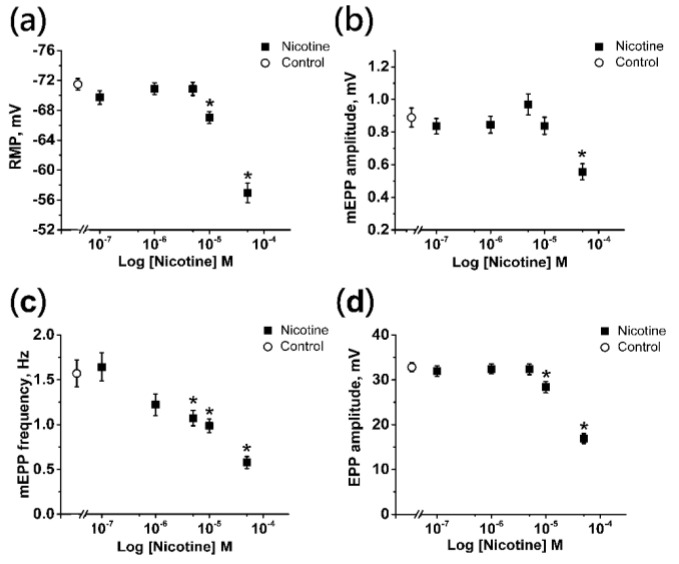
Effects of nicotine on the electrophysiological parameters registered at the mouse neuromuscular junction. Changes in absolute values are shown (**a**) resting membrane potential (RMP) of muscle fibers, (**b**) amplitudes of miniature endplate potentials (mEPP), (**c**) frequency of the mEPPs, and (**d**) the amplitudes of evoked endplate potentials (EPP) in the control and 15 min after nicotine application (the range from 0.1 to 50 μM). Results are expressed as mean ± SEM. Asterisks (*) indicate significant effects (*p* < 0.05, one-way ANOVA test with Dunnet’s post-hoc comparison).

The control RMP value of muscle fibers was −71.48 ± 0.77 mV (*n* = 30). Application of nicotine at concentrations of 0.1 μM, 1 and 5 μM did not affect the RMP significantly, providing values of −69.72 ± 0.90 mV (*n* = 30), −70.91 ± 0.78 mV (*n* = 30), and −70.85 ± 0.87 mV (*n* = 30), respectively (Figure 1a). A slight significant depolarization was observed when nicotine concentration was increased to 10 μM (−67.04 ± 0.79 mV; *n* = 30). At a concentration of 50 μM, a more pronounced depolarization was observed, and the mean RMP value decreased to −56.94 ± 1.29 mV (*n* = 30; Figure 1a).

Another sign of the postsynaptic action of nicotine was the change in the amplitude of mEPP. The mean value of the amplitude of the spontaneous signal in the control was 0.88 ± 0.05 mV (*n* = 30). Application of nicotine in concentration up to 10 μM did not affect mEPP amplitude significantly, with the values being 0.83 ± 0.04 mV (*n* = 30) for 0.1 μM, 0.84 ± 0.05 mV (*n* = 30) for 1 μM, 0.96 ± 0.06 mV (*n* = 30) for 5 μM, and 0.83 ± 0.05 mV (*n* = 30) for 10 μM (Figure 1b). Asignificant decrease in mEPP amplitude to 0.55 ± 0.04 mV (*n* = 30) was observed only with 50 μM nicotine (Figure 1b).

In contrast to the amplitude of mEPPs, the effect of nicotine on the frequency of occurrence of spontaneous signals was detected at significantly lower concentrations. That is, the average values of the frequency of mEPPs upon application of 0.1 and 1 μM nicotine were 1.64 ± 0.15 Hz (*n* = 30) and 1.22 ± 0.12 Hz (*n* = 30), respectively, and did not differ from the control value of 1.57 ± 0.14 Hz (*n* = 30; Figure 1c). After application of 5 μM nicotine, the frequency significantly decreased to 1.07 ± 0.08 Hz (*n* = 30), and inhibition was further enhanced to 0.98 ± 0.07 Hz (*n* = 30) for 10 μM and 0.57 ± 0.06 Hz (*n* = 30) for 50 μM (Figure 1c).

The amplitude of the EPP, which reflects the level of evoked ACh release and depends on changes in the sensitivity of the postsynaptic membrane in the area of the neuromuscular contact, was 32.80 ± 1.02 mV (*n* = 30) in the control. Application of nicotine at concentrations of 0.1 μM, 1 and 5 μM did not alter the average amplitudes of EPP, which were equal to 31.93 ± 1.21 mV (*n* = 30), 32.43 ± 1.11 mV (*n* = 30), and 32.33 ± 1.18 mV (*n* = 30), respectively (Figure 1d). However, nicotine produced a decrease in EPP amplitude, starting at the concentration of 10 μM (28.36 ± 1.27 mV; *n* = 30), while at 50 μM the amplitude decreased almost twofold to 16.89 ± 1.09 mV (*n* = 30; Figure 1d).

Thus, for further investigations of the ACh release autoregulation mechanisms, concentration of 10 μM nicotine was chosen. When using nicotine at this concentration, a decrease in the EPP amplitude was observable, while there were no changes in the mEPP amplitude (with only a slight depolarization of the sarcolemma).

### 2.2. Activation of Neuronal Nicotinic Receptors Leads to Downregulation of the EPP Quantal Content

**Figure 2:**
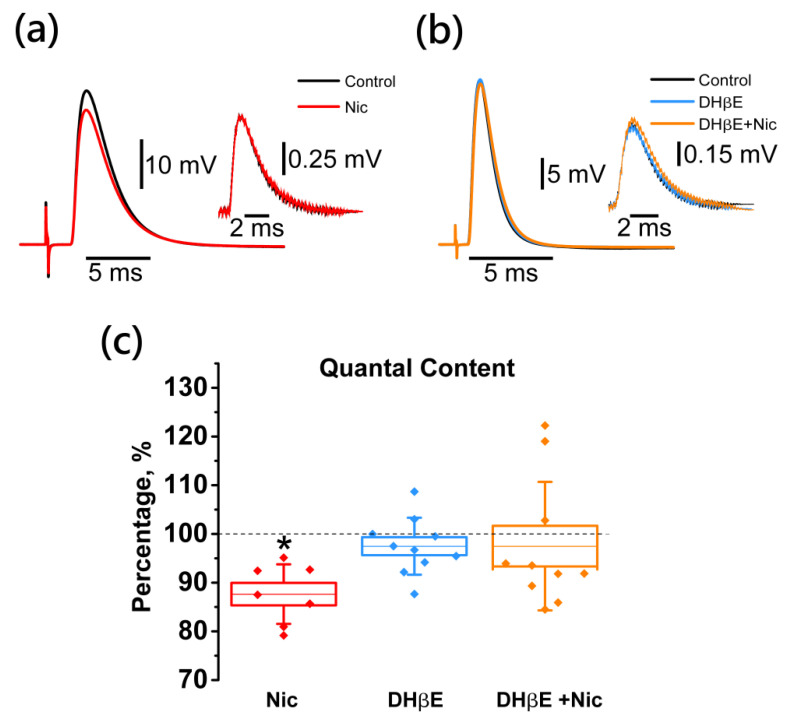
Nicotine inhibits the evoked release of ACh quanta (quantal content, QC) by activating nNAChRs. Panels on the top are representative traces of EPP and mEPP (50 signals averaged) in separate experiments with nicotine application (Nic, 10 µM; (**a**)), and nicotine application after pretreatment with the neuronal cholinergic receptor antagonist DHβE (1 µM; (**b**)). (**c**) Results are expressed as mean ± SEM and SD of QC, as percentages with nicotine (*n* = 7), DHβE (*n* = 9), and DHβE plus nicotine (*n* = 9) applications versus control. Asterisks (*) indicate significant effects (*p* < 0.05, one-way repeated ANOVA test with Tukey’s post-hoc comparison).

Under control conditions, the quantal content (QC) was 46.8 ± 4.5. The bath application of nicotine (10 µM) decreased the QC of EPP significantly by 12.0 ± 4.4% (*n* = 7; Figure 2).

The nicotine-induced decrease in the number of ACh quanta released in response to stimulation of the motor nerve suggests the involvement of presynaptic cholinergic receptors. Using the antagonist of neuronal nicotinic ACh receptors (nNAChRs) DHβE [27,28], we obtained the data supporting this suggestion. The application of DHβE alone at a concentration of 1 μM did not change the QC of EPP (101.5 ± 1.3%; *n* = 9, Figure 2), however, after pretreatment with DHβE, the inhibitory effect of nicotine on the quantal release of ACh was completely abolished (105.5 ± 6.6%; *n* = 9; Figure 2).

### 2.3. Activation of Neuronal Nicotinic Receptors Induces an Increase of the Calcium Level in the Motor Nerve Terminal

Since the process of the evoked release of a neurotransmitter is triggered by the entry of calcium ions into the nerve ending [29,30], it was suggested that the inhibitory effect of nicotine on ACh release could be related to a decrease in Ca^2+^ influx.

**Figure 3:**
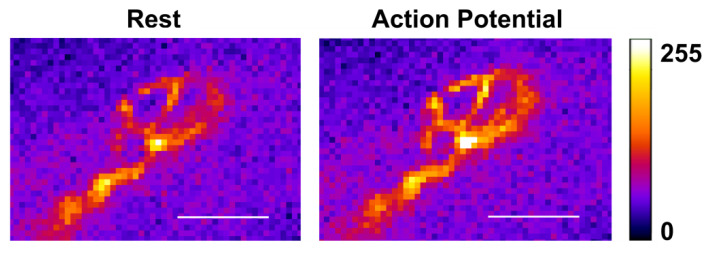
Pseudo-color calcium images of a motor nerve terminal loaded with Oregon Green 488 BAPTA-1 Hexapotassium Salt. The axon is imaged before and during single electrical stimulus (0.2 ms duration). Scale bar, 20 µm.

**Figure 4:**
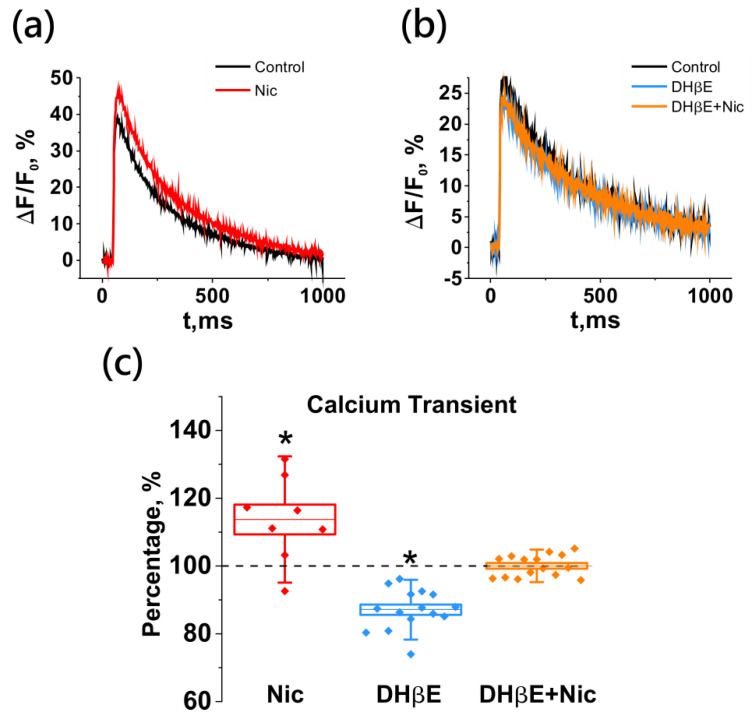
Nicotine increases the calcium transient in the motor nerve ending by the activation of neuronal ACh receptors. Blockade of the receptors leads to a decrease in the amplitude of the calcium signal. Panels on the top are representative traces of calcium transient from separate experiments with nicotine application (Nic, 10 µM; (**a**)), and nicotine application after pretreatment with an nNAChR antagonist DHβE (1 µM; (**b**)). (**c**) Mean ± SEM and SD of the amplitude of the calcium signal, expressed as a percentage of control when applying nicotine (*n* = 8), DHβE (*n* = 15) and DHβE plus nicotine (*n* = 15). Asterisks (*) indicate significant effects (*p* < 0.05, one-way repeated ANOVA test with Tukey’s post-hoc comparison).

The amplitude changes of the optical signal (ΔF/F_0_) from the calcium dye loaded into the nerve terminal in response to a single stimulus (with the same characteristics as during EPP registration) averaged about 30% (Figure 3). Nicotine application did not lead to a decrease, as expected, but instead caused a significant increase in the amplitude of the calcium transient by 13.7 ± 4.3% (*n* = 8; Figure 4). Thus, in the presence of a nicotinic receptor agonist, the presynaptic calcium level increases more strongly in response to nerve stimulation than in its absence. Is this increase indeed triggered by nNAChRs, whose activation leads to a decrease in the subsequent ACh release? The answer to this question was obtained in the experiments with an antagonist of nicotinic receptors, DHβE.

Application of the antagonist alone led to a decrease in the calcium transient significantly by 12.9 ± 1.5% (*n* = 15; Figure 4). However, after pretreatment with DHβE, the calcium signal-enhancing effect of nicotine was completely abolished (100.0 ± 0.8%; *n* = 15; Figure 4).

### 2.4. Neuronal Nicotinic Receptors Alter Calcium Level in Presynaptic Terminal by Gating L-Type (Ca_v_1) Calcium Channels

**Figure 5:**
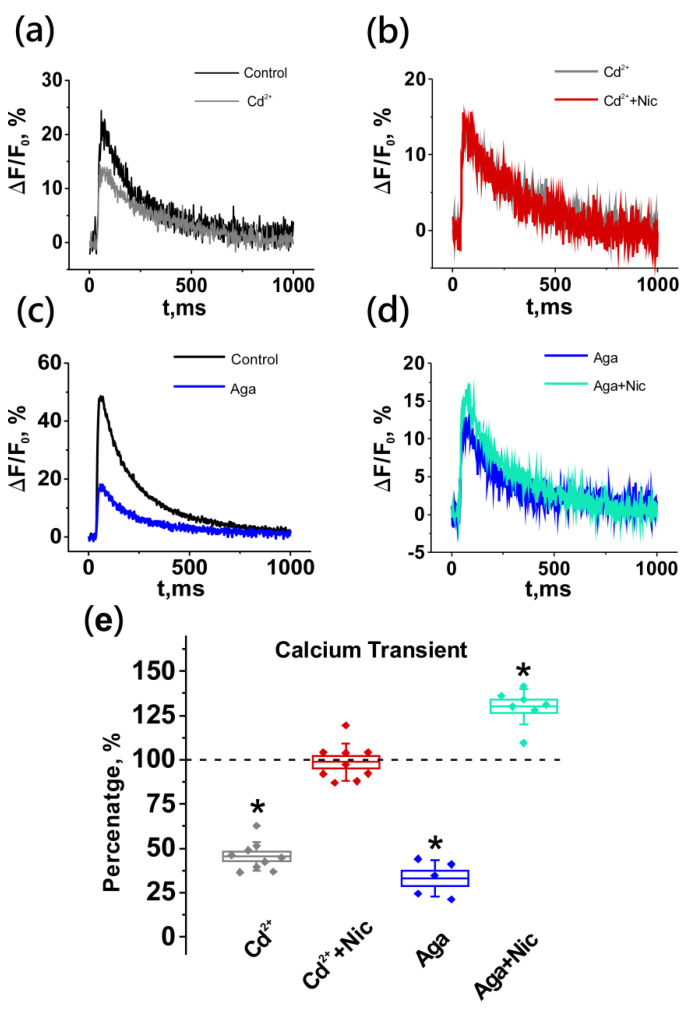
The calcium transient-enhancing effect of nicotine is abolished after a nonspecific calcium channel blockade, but not after inhibition of Ca_v_2.1-type calcium channels. Panels on the top are representative traces of calcium transient from individual experiments: (**a**) the effect of the nonspecific calcium channel blocker CdCl_2_ (Cd^2+^, 10 µM), (**b**) no effect of nicotine (Nic, 10 µM) after pretreatment with CdCl_2_, (**c**) the effect of the Ca_v_2.1 VGCC blocker ω-agatoxin IVA (Aga, 40 nM), and (**d**) the effect of nicotine on the calcium transient after pre-incubation with ω-agatoxin IVA. (**e**) Mean ± SEM and SD of calcium signal amplitudes obtained in the above series and expressed as a percentage of control or value after CdCl_2_ (*n* = 9), CdCl_2_ + Nic (*n* = 9), Aga (*n* = 5;) and Aga + Nic (*n* = 7) application. Asterisks (*) indicate significant effects (*p* < 0.05, one-way repeated ANOVA test).

To identify the source of the increase in the calcium signal upon the activation of presynaptic nNAChRs, cadmium chloride, a nonselective blocker of calcium-permeable channels, was used at a concentration of 10 µM. After the application of cadmium chloride, a decrease in the amplitude of the calcium transient was observed by 54.5 ± 2.7% (*n* = 9). In the presence of cadmium, the effect of nicotine on the alterations in calcium levels was completely abolished (101.4 ± 3.4%; *n* = 9; Figure 5). Therefore, the observed increase in the presynaptic calcium level upon the activation of nNAChRs is mediated by proteins (channels) which are permeable for Ca^2+^. Further experiments were carried out to establish which type of VGCCs are involved in nicotine-induced increases in calcium transients.

The application of the specific P/Q-type (Ca_v_2.1) VGCCs blocker ω-agatoxin IVA at a concentration of 40 nM that blocks only a certain proportion of channels [31] led to a significant decrease in the calcium transient by 67.0 ± 4.4% (*n* = 5; Figure 5). In the case of a partial blockade of the main type of VGCCs Ca_v_2.1, nicotine application (10 µM) led to an increase in the amplitude of the calcium transient significantly by 29.9 ± 3.8% (*n* = 10, Figure 5). Therefore, the effect of the activation of nNAChRs on the intracellular calcium level is not mediated by Ca_v_2.1 channels.

**Figure 6:**
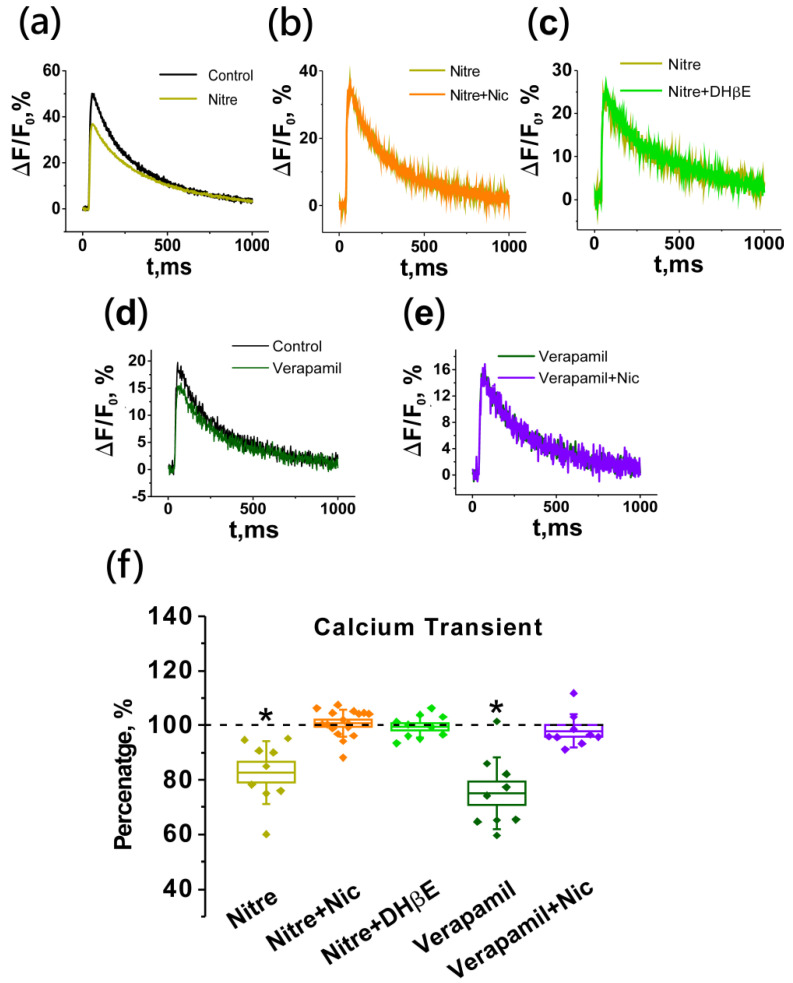
Lack of effect of nicotine (an increase in the amplitude of the calcium transient) and DHβE (a decrease in the amplitude of the calcium transient) after blockade of the Ca_v_1 channels. Panels on the top are representative traces of calcium transient from individual experiments: (**a**) effect of Ca_v_1 calcium channel blocker nitrendipine (Nitre, 25 µM), (**b**) lack of nicotine (Nic, 10 µM) effect after pre-application of nitrendipine, (**c**) lack of DHβE (1 µM) effect after nitrendipine pre-treatment, (**d**) effect of Ca_v_1 type VGCCs blocker verapamil (50 µM), (**e**) no effect of nicotine after pre-application of verapamil. (**f**) Mean ± SEM and SD of calcium signal amplitudes obtained in the above series and expressed as a percentage of control or value after Nitre (*n* = 17), Nitre plus Nic (*n* = 17), Nitre plus DHβE (*n* = 7), verapamil (*n* = 9) and verapamil plus Nic (*n* = 9) application. Asterisks (*) indicate significant effects (*p* < 0.05, one-way repeated ANOVA test).

Ca_v_1 calcium channel blockers such as verapamil (50 µM) and nitrendipine (25 µM), produced significant calcium transient decreases of 25.0 ± 4.4% (*n* = 9) and 18.8 ± 1.1% (*n* = 17), respectively (Figure 6). The application of nicotine after pretreatment by these blockers did not cause any changes in the calcium transient: the amplitudes were 101.8 ± 1.5% (*n* = 9) and 100.7 ± 1.2% (*n* = 17), respectively (Figure 6).

These data allow us to conclude that the observed increase in the calcium level in the nerve ending upon activation of nNAChRs by an exogenous agonist is due to mediation by Ca_v_1 type VGCCs. Therefore, the phenomenon of the endogenous activation of presynaptic cholinergic receptors discovered by us should also be mediated by calcium channels of this type. Indeed, the calcium transient-reducing effect of DHβE, when Ca_v_1 channels were blocked by nitrendipine, was completely abolished (100.0 ± 0.8%; *n* = 7; Figure 6).

Thus, the results obtained allow us to conclude that activation of nNAChRs leads to an additional increase in the entry of Ca^2+^ into the nerve ending through VGCCs of the Ca_v_1 type. Therefore, if this is the mechanism underlying the decrease in the quantal content upon activation of this type of cholinergic receptor, then it should be expected that the blockade of Ca_v_1 type of calcium channels will eliminate the nicotine-induced decrease in the amount of released ACh quanta. Examining this assumption became the scope of the next step of the study.

### 2.5. Nicotine-Induced Decrease in Acetylcholine Release Is Mediated by L-Type (Ca_v_1) Calcium Channels and Not Coupled to Apamin-Sensitive K_Ca_ Channels

To assess the possible role of Ca_v_1 type of calcium channels in the nicotine-induced mechanism of ACh release autoinhibition, verapamil and nitrendipine were used at the same concentrations as in experiments with calcium transients.

**Figure 7:**
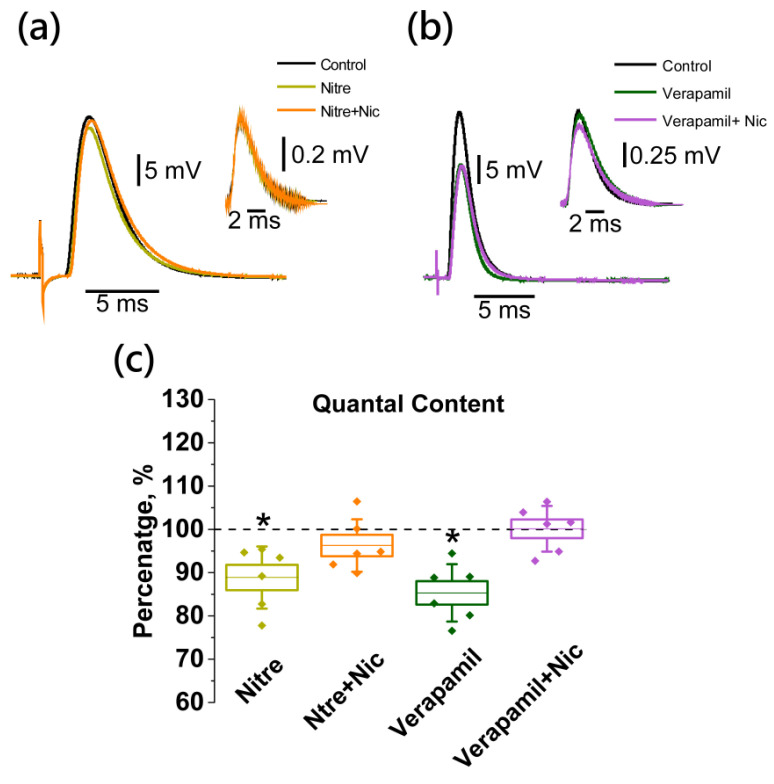
Nicotine-induced decrease in the ACh release (quantal content, QC) involves L-type Ca_v_1 channels. Panels on the top are representative traces of EPP and mEPP (50 signals averaged) from individual experiments: (**a**) and (**b**) lack of nicotine (Nic, 10 µM) effect after pre-application with the Ca_v_1 VGCC blockers nitrendipine (Nitre, 25 µM) and verapamil (50 µM); (**c**) mean ± SEM and SD of QC, expressed as a percentage of control or value after Nitre (*n* = 6), Nitre plus Nic (*n* = 6), Verapamil (*n* = 6) and Verapamil plus Nic (*n* = 6) application. Asterisks (*) indicate significant effects (*p* < 0.05, one-way repeated ANOVA test with Tukey’s post-hoc comparison).

Verapamil and nitrendipine application resulted in a significant decrease in the QC by 14.2 ± 3.2% (*n* = 6) and 11.2 ± 2.9% (*n* = 6), respectively (Figure 7). Nicotine application after pre-treatment with Ca_v_1 channel blockers did not cause any changes in the evoked ACh release, and the QCs were 101.6 ± 1.9% (*n* = 6) and 103.8 ± 2.4% (*n* = 6), respectively (Figure 7).

One of the possible calcium-activated targets that may be responsible for the nicotine-induced depression of ACh release is the apamin-sensitive K_Ca_ channel [21]. To examine the involvement of this channel into the nicotine-induced decrease of ACh release from the motor nerve terminal, experiments with K_Ca_ blocker apamin were conducted.

**Figure 8:**
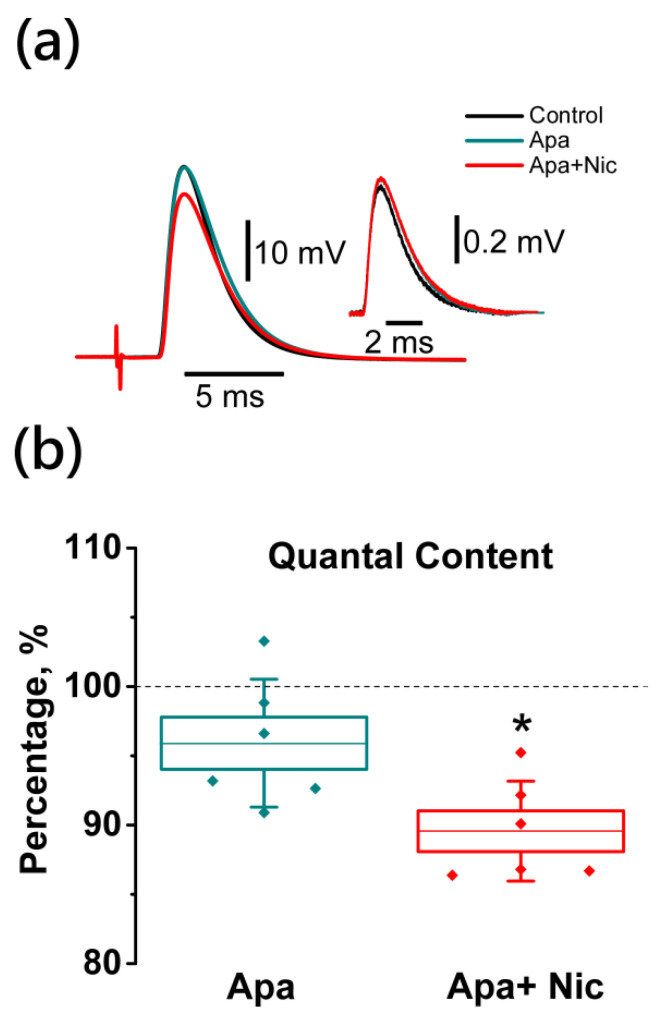
Nicotine-induced decrease in ACh release (quantal content, QC) is observed in the presence of apamin (Apa), the blocker of small conductance Ca^2+^-activated K^+^ channels. Panel on the top (**a**) is representative traces of EPP and mEPP (50 signals averaged) from individual experiments with apamin application (100 nM) and with nicotine (Nic, 10 μM) application after pretreatment with apamin; (**b**) Mean ± SEM and SD of QC, expressed as a percentage of control or value after apamin (*n* = 6), apamin + Nic (*n* = 6). Asterisks (*) indicate significant effects (*p* < 0.05, one-way repeated ANOVA test with Tukey’s post-hoc comparison).

The application of this blocker to small-conductance Ca^2+^-activated K^+^ channels alone at a concentration of 100 nM did not change the QC of EPP (95.9 ± 1.8%; *n* = 6, Figure 8). In the presence of apamin the effect of nicotine on the QC of EPP remained unchanged (10.5 ± 1.4%; *n* = 6, Figure 8). Therefore, the effect of the activation of nNAChRs on the quantal release of ACh is mediated by Ca_v_1 channels and not coupled to apamin-sensitive K_Ca_ channels.

## 3. Discussion

### 3.1. Effects of Nicotine on ACh Release

The results of our study demonstrate that in the mouse neuromuscular preparation of m.LAL, nicotine at concentrations up to 1 μM has no effect on either the processes of ACh release from the nerve terminal or on the processes of its interaction with the postsynaptic membrane. At the concentration of 5 μM, the presynaptic effect of nicotine appears to become detectable (inhibition of the spontaneous release of ACh due to the activation of presynaptic cholinergic receptors). An increase in concentration to 10 μM enhances the presynaptic effect of the alkaloid (suppression of not only spontaneous, but also of the evoked ACh release) and leads to a weak postsynaptic effect (decrease in RMP due to the activation of postsynaptic cholinergic receptors). With an increase in nicotine concentration to 50 μM, dramatic changes become evident in all recorded parameters of neurotransmission.

Thus, nicotine at a concentration of 10 μM exerts both postsynaptic and presynaptic inhibitory effects on the neuromuscular synapse, causing a decrease in the number of ACh quanta released in response to action potential. A similar decrease in the QC during the activation of cholinergic receptors has been noted earlier [17,20,21,32], however, these results were obtained on other preparations, in conditions of initially reduced QC, or in cut fiber preparations.

### 3.2. Presynaptic Cholinergic Receptors and the Role of Calcium Influx in the Mechanism of ACh Release Autoinhibition

Since the inhibitory effect of nicotine on the QC was completely abolished by the application of the antagonist of neuronal cholinergic receptors DHβE, it was concluded that these receptors are involved in the cholinergic mechanism of regulation of ACh release. DHβE binds to the β2 subunits of neuronal receptors and is a selective antagonist for non-α7 nAChRs [33]. In the heteromeric receptors of ganglionic neurons the primary α subunit is α3, whereas in the rodent central nervous system the primary α subunit is α4 [34]. The α4β2 nNAChR is the most abundant subtype expressed in the brain, and studies have demonstrated that this receptor subtype is located presynaptically [35]. Therefore, we can assume that, in the neuromuscular synapse, neuronal cholinergic receptors have the α4β2 subunit composition. This assumption is supported by the data that at 1 μM DHβE, that we used in this study, the mouse α4β2 receptors are almost completely blocked, while the α3β4 subunit receptors remain essentially active [34].

In the next step of the mechanism of autoinhibition triggered by nNAChRs, it was necessary to answer the following key question: how is the activation of the presynaptic nicotinic receptors coupled to changes in the intracellular calcium level? Previous data [20,32,36] were indicating such a coupling, but there was no direct evidence found for this prior to our study. Using standard electrophysiological methods, combined with the fluorescent method for registration of calcium transients, which reflect changes in the calcium level within the presynaptic terminal upon action potential arrival, enabled us to obtain data on changes in the ACh release. Our results demonstrate that the activation of nNAChRs (sensitive to DHβE), leading to a decrease in ACh release, is accompanied by an increase in the level of calcium in the nerve terminal. Another important observation made was the significant effect of DHβE on the amplitude of the calcium transient when applied alone. This may indicate that there exists a background tonic activation of nNAChRs which results in a tonic increase in calcium entry into the nerve terminal. We suggest that the release of endogenous ACh during motor nerve stimulation and the spontaneous quantal release under physiological conditions results in the modulation of presynaptic Ca^2+^ entry and provides a physiologically important feedback [17]. In the absence of impulse activity, the largest amount of ACh is released from the nerve terminal through non-quantal release [37]. This ACh is able to tonic the activation of muscarinic cholinergic receptors at the neuromuscular synapse [38]. Therefore, it can be assumed that DHβE-sensitive nicotinic cholinergic receptors can also be activated by a non-quantal ACh.

The complete absence of the effect of nicotine on the calcium signal after pre-treatment with cadmium (10 μM), which is a nonselective blocker of all types of calcium Ca_v_ channels [39] allows two conclusions to be put forward: (i) the increase in the calcium level in the nerve terminal is mediated by transmembrane proteins permeable for Ca^2+^ from the environment; (ii) the observed entry of Ca^2+^ is mediated by channels other than those of nNAChRs. The last suggestion is very important, because it has been shown earlier that nNAChRs are more permeable to Ca^2+^ as compared to permeability of their muscle-type counterparts [40,41]. At the same time, it was shown that cadmium up to a concentration of 200 μM does not block currents through nNAChRs [42], but significantly blocks currents through VGCCs [43,44].

After the inactivation of VGCCs of Ca_v_2.1 type, which are key to triggering the process of evoked ACh release [31,45,46] the effect of nicotine on calcium entry into the terminal was preserved, while after the blockade of Ca_v_1 type channels, it was completely abolished. It should be noted that the possibility of the involvement of these channels in the evoked release of ACh quanta remained under debate until recently, as in [20,47,48,49] versus [23,24,25,26]. We have obtained clear evidence of the involvement of the Ca_v_1 type of calcium channels in the regulation of the bulk calcium level in the terminal, and of the process of neurotransmission in the mammalian neuromuscular junction.

### 3.3. Critical Issues in Establishing the Coupling between nNAChRs and L-Type (Ca_v_1) Calcium Channels while Using a Pharmacological Approach

The phenylalkylamine (verapamil), dihydropyridine (nitrendipine) and benzothiazepine classes of Ca_v_1 type calcium channel blockers are capable of blocking nNAChRs [42,50]. The absence of the effects of nicotine (both on the calcium transient and on the QC) after the application of verapamil and nitrendipine may well be related to a simple direct blockade of nNAChRs (sensitive to DHβE and permeable to Ca^2+^). Indeed, DHβE, verapamil, and nitrendipine all lead to a decrease in the transients. At the same time, all three pharmacological agents abolish the effect of nicotine.

If even the direct blockade of nNAChRs by verapamil and nitrendipine does take place, a number of additional facts still point to the involvement of the Ca_v_1 calcium channels in the mechanism of modulation of calcium entry and the process of ACh release in the nerve terminal. So, after the application of all three agents, the calcium entry decreases, however, the effect of verapamil and nitrendipine is almost two times more pronounced than that of DHβE. Furthermore, the QC does not change after the addition of DHβE, whereas in the presence of verapamil and nitrendipine it is decreased by more than 10%. In addition, the activation of cholinergic receptors (by nicotine) and presumed blockade of cholinergic receptors (by verapamil and nitrendipine) have not an opposite, but a unidirectional effect: a decrease in the QC. And, last but not least, the absence of the effect of nicotine on the calcium transient after the application of cadmium, which does not affect the functioning of nNAChRs (including α4β2 nNAChRs) or even potentiate them [42,51], indicates that in our case a pharmacological effect on two different targets took place.

### 3.4. How the Activation of nNAChRs Modulates L-Type (Ca_v_1) Channel Functioning

**Figure 9:**
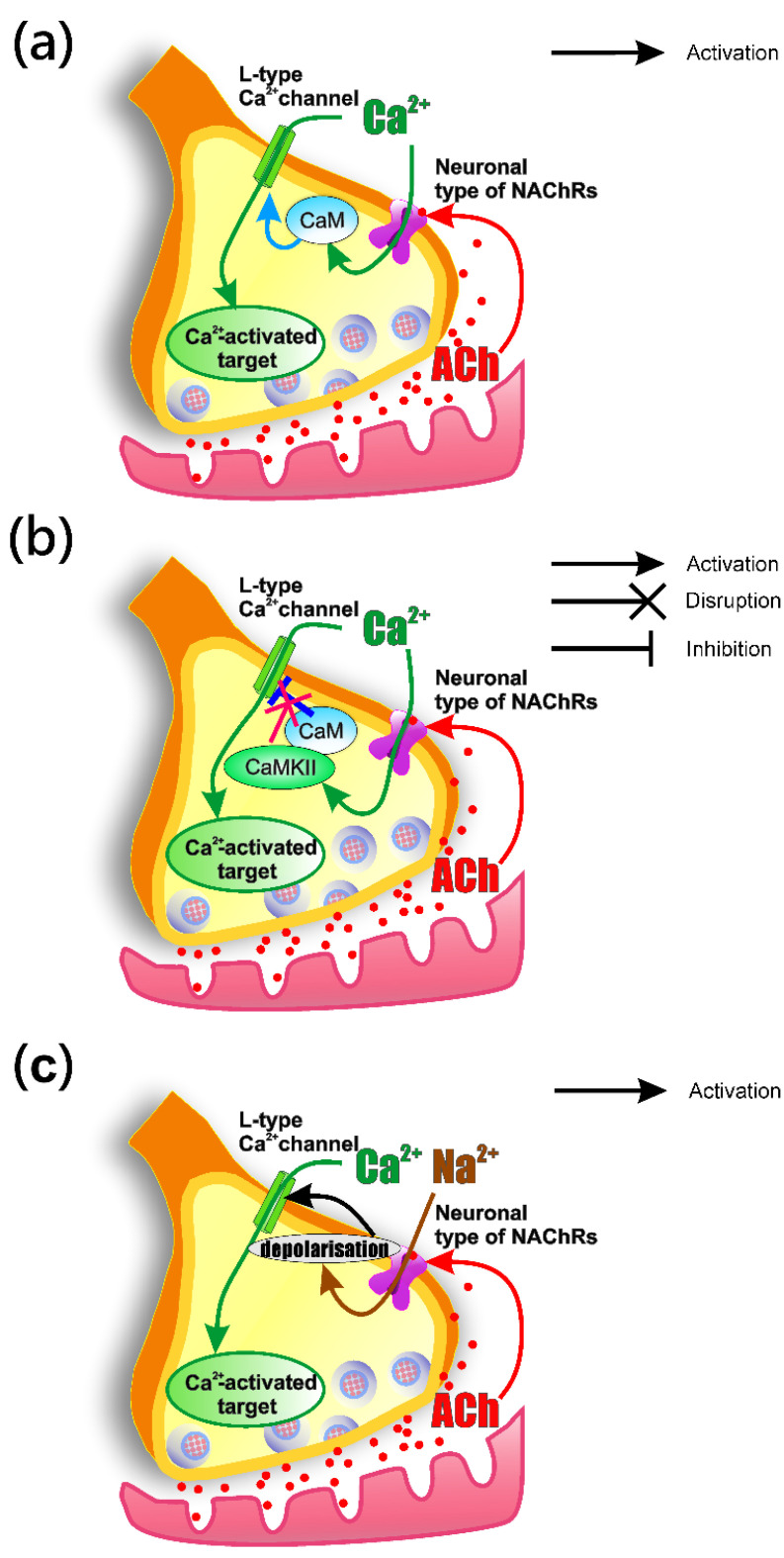
Schematic drawing showing possible mechanisms of the coupling between nAChRs and L-type (Ca_v_1) calcium channels in the motor nerve terminal. (**a**) Calcium dependent facilitation of the Ca_v_1 type channel, mediated by CaM and triggered by calcium [52], which enters through nNAChRs; (**b**) calcium dependent inactivation process of Ca_v_1 type calcium channels, mediated by the interaction between CaM and the Ca^2+^ channel, is disrupted by an increase in CaMKII activity [53]; (**c**) opening of Ca_v_1 calcium channels and entry of calcium into terminal caused by depolarization due to activation of nAChRs [54].

nNAChRs have high Ca^2+^ permeability [41], which does not have a direct effect on the calcium transient amplitude. However, calcium entry through these receptors can lead to two potential outcomes and one can observe an increase in the amplitude of the calcium transient as a result of two mechanisms: (i) the CDF process (calcium dependent facilitation) of the Ca_v_1 type channel is triggered [52] (Figure 9a); (ii) the CDI (calcium dependent inactivation) process of Ca_v_1 type calcium channels, mediated by the interaction between CaM and Ca^2+^ channel, is disrupted by increasing CaMKII activity [53] (Figure 9b). However, according to [20], a decrease in ACh release caused by activation of nNAChRs is not associated with CaM.

The existence of a functional interaction between nNAChRs and the channels of the Ca_v_1 type was established in a primary culture of neurons in the mouse cerebral cortex [54]. It should be noted that this work revealed the interaction of calcium channels with α4β2 nNAChRs. Potentially, a similar interaction takes place in the muscle-nerve junction. The authors believe that activation of presynaptic receptors leads to depolarization sufficient for opening of Ca_v_1 calcium channels and entry of calcium into the neuron [54] (Figure 9c).

The question that arises is how enhanced Ca^2+^ entry via Ca_v_1 channels can result in the downregulation of the ACh release. Firstly, the results obtained on the neuromuscular preparations suggest that the calcium channels of Ca_v_1 type are located far from the active zones [55], an therefore that Ca^2+^ entering through them cannot directly interact with the exocytosis machine. Secondly, it was shown repeatedly that the elevation of the intra-terminal Ca^2+^ level could activate calcium-sensitive proteins participating in the downregulation of neurotransmitter release. Among them, the most probable candidates are calmodulin, calcineurin, and Ca^2+^-activated K^+^ (K_Ca_) channels [21,56,57]. However, according to our data, apamin-sensitive K_Ca_ channels are not involved into the nicotine-induced effects on the evoked ACh release.

## 4. Materials and Methods

### 4.1. Animals

Mice BALB/C (20–23 g, 2–3 months old) of either sex were used in this study. All animal care and experimental protocols met the requirements of the European Communities Council Directive 86/609/EEC and was approved by the Ethical Committee of Kazan Medical University (No. 10; 20 December 2016). Animals were housed in groups of 10 animals divided by gender inside plastic cages with plenty of food (standard mice chow) and water ad libitum. The temperature (22 °C) of the room was kept constant and a 12 h light/dark cycle was imposed. Animal studies are reported in compliance with the ARRIVE guidelines [58] and with the recommendations made by the British Journal of Pharmacology [59].

### 4.2. Tissue Preparations and Solutions

Animals were euthanized by cervical dislocation in accordance with the approved project protocol. The *Levator auris longus* muscle (m. LAL) was quickly removed [60], then put into a Sylgard^®^ chamber with bubbled (95% O_2_ and 5% CO_2_) Ringer solution (pH 7.4) containing (in mM): NaCl 137, KCl 5, CaCl_2_ 2, MgCl_2_ 1, NaH_2_PO_4_ 1, NaHCO_3_ 11.9, and glucose 11. Bath solution temperature was controlled by a Peltier semiconductor device. Experiments were performed at 20.0 ± 0.3 °C. Muscle contractions were prevented using µ-conotoxin GIIIB in a 2 µM concentration [61].

### 4.3. Electrophysiology

We used a standard intracellular recording technique [14,62]. Microelectrodes were prepared from borosilicate glass (World Precision Instruments, Sarasota, FL, USA) using a P97 micropipette puller (Sutter Instrument, Novato, CA, USA). Recording electrodes were 20–30 MΩ and filled with 3 M KCl. To record evoked and spontaneous (miniature) endplate potentials (EPPs and mEPPs, respectively) an Axoclamp 900 A amplifier and a DigiData 1440 A digitizer (Axon Instruments, San Jose, CA, USA) were used. Membrane potential was at −60 to −80 mV and recorded by a miniDigi 1B (Axon Instruments, San Jose, CA, USA). Experiments with membrane potential deviations over 7 mV were declined. The nerve was stimulated with rectangular suprathreshold stimuli (0.2 ms duration, 0.5 Hz frequency) via a suction electrode connected to a model 2100 isolated pulse stimulator (A-M Systems, Sequim, WA, USA). All electronic devices were driven by pClamp v10.4 software (Molecular Devices, San Jose, CA, USA). Bandwidth was from 1 Hz to 10 kHz. After collecting 35 EPPs, mEPPs during 2 min were recorded. Quantal content was estimated as ratio of averaged EPPs to mEPPs amplitude.

### 4.4. Calcium Transient Recording

Nerve motor endings were loaded with Oregon Green 488 BAPTA-1 Hexapotassium Salt 1 mM high-affinity calcium-sensitive dye (Molecular Probes, Eugene, OR, USA) through the nerve stump, as described previously [44]. The fluorescence signal was recorded using an imaging setup based on an Olympus BX-51 microscope with a ×40 water-immersion objective (Olympus, Tokyo, Japan). Calcium transient registration was performed via a high-sensitivity RedShirtImaging NeuroCCD-smq camera (RedShirtImaging, Decatur, GA, USA), at 500 fps (exposure time 2 ms) and 80 × 80 pixels, which was sufficient for calcium transient registration with good temporal resolution. As the source of light, a Polychrome V (Till Photonics, Munich, Germany) was used with a set light wavelength of 488 nm. The following filter set was used to isolate the fluorescent signal: 505DCXT dichroic mirror, E520LP emission (Chroma, Bellows Falls, VT, USA). We used Turbo-SM software (RedShirtImaging, Decatur, GA, USA) for data recording. In each experiment, 8 fluorescence responses were recorded, then averaged. This was an optimal amount to obtain data of sufficient quality and to reduce excitotoxicity and photobleaching of the fluorophore.

To analyze the recorded images, ImageJ software (NIH, Bethesda, MD, USA) was used. We picked regions of interest in the motor nerve ending image and background manually. Subsequent data processing was performed in Excel (Microsoft, Redmond, WA, USA). Background values were averaged and subtracted from signal values. Data were represented as a ratio: (ΔF/F_0_ − 1) × 100 %, where ΔF is the fluorescence intensity during stimulation, and F_0_ is the fluorescence intensity at rest [63].

### 4.5. Materials

Nicotine hydrogen tartrate salt (nicotine), nitrendipine, verapamil, ω-agatoxin IVA, cadmium chloride, and dimethylsulfoxide (DMSO) were obtained from Sigma Aldrich (St. Louis, MO, USA). Dihydro-β-erythroidine hydrobromide (DHβE), apamin (TOCRIS, Bristol, UK), and µ-conotoxin GIIIB were obtained from Peptide Institute Inc. (Osaka, Japan). Drugs were dissolved in distilled water with the exceptions of nitrendipine and verapamil, which were dissolved in DMSO. Further dilutions for all drugs were done in Ringer solution. In experiments with drugs which were dissolved in DMSO, the same concentration of DMSO was added to the control solution as was present in the solution with the agent. Finally, the DMSO concentration in the solution did not exceed 0.01%.

### 4.6. Data and Statistical Analysis

Data collection and statistical analysis comply with the recommendations of the British Journal of Pharmacology on experimental design and analysis in pharmacology [64]. The number of experiments in each experimental group was selected on the basis of observing a statistically significant effect while using the minimum number of animals (3R principles) and on experience from previous studies. Animals were randomly assigned to the different experimental groups, with each group having the same number of animals by design. Blinding of the operator was not feasible, but data analysis was performed semi-blinded by an independent analyst.

Statistical analysis was performed using Statistica 6.1 Base (Tulsa, OK, USA). A Shapiro–Wilk normality test was used to analyze the data distribution. Null-hypothesis testing was performed by ANOVA. One-way ANOVA followed by Dunnett’s or Tukey’s test for multiple comparison post hoc was used. For related groups, a one-way repeated ANOVA test was performed. Data are presented as mean ± SEM. Values of *p* < 0.05 were considered significant.

## 5. Conclusions

**Figure 10:**
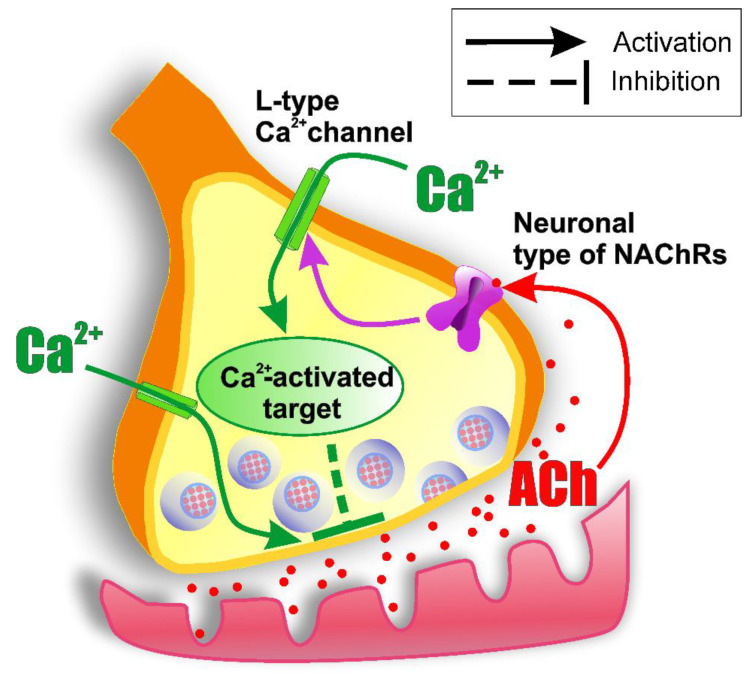
Working model of the mechanism of autoregulation of ACh release in the peripheral cholinergic synapse via nNAChRs. Activation of nNAChRs is accompanied by an increase in the entry of calcium ions into the motor nerve terminal through the L-type (Ca_v_1) calcium channels. The latter are involved in both the process of evoked ACh release and its modulation.

In the present study, we found that activation of presynaptic nNAChRs leads to a decrease in the quantal ACh release from the nerve ending. This negative feedback mechanism is mediated by the modulating of the function of VGCCs of the Ca_v_1 type, which leads to an increase in the entry of Ca^2+^ into the nerve terminal (Figure 10). Understanding of the peculiarities of action of ACh (nicotine) on the nNAChR-containing nerve endings (not only in cholinergic synapses [51,65]) has a broad scientific and clinical significance, since cholinergic nicotinic signaling (in addition to neuromuscular transmission and synaptic transmission in ganglia) is involved in the setting of a variety of processes, including anxiety, depression, arousal, memory, and attention [66,67].

## Data Availability

The data that support the findings of this study are available from the corresponding author upon reasonable request.

## Abbreviations

ACh	acetylcholine
VGCCs	voltage-gated calcium channels
RMP	resting membrane potential
mEPPs	miniature endplate potentials
EPPs	end plate potentials
QC	quantal content
nNAChRs	neuronal nicotinic ACh receptors
DHβE	dihydro-β-erythroidine hydrobromide
